# Supplementary material for: A Global Screen for Assembly State Changes of the Mitotic Proteome by SEC-SWATH-MS
Source: Cell Syst. 2020 Feb 26;10(2):133–155.e6. doi: 10.1016/j.cels.2020.01.001 (PMC7042714; doi:10.1016/j.cels.2020.01.001)

Q8WYA6 | CTBL1_HUMAN | CTNNBL1 C20orf33 PP8304
Monomer MW [kDa]: 65.173 Monomer expected elution fraction: 44

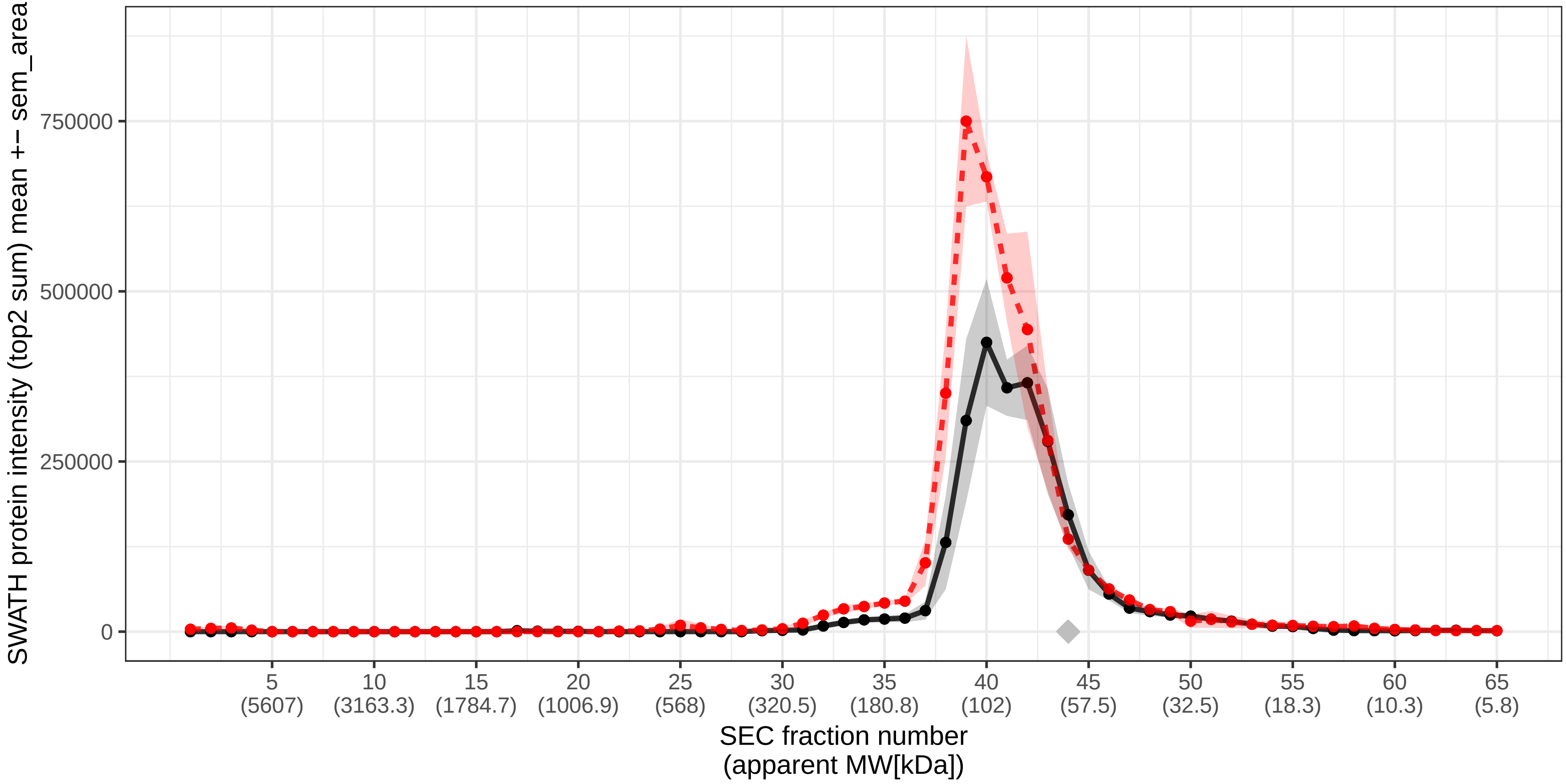

Supplement: Data S1. SEC-SWATH-MS Protein Chromatograms, Related to Figure 1 [file mmc6.zip › SECchrom_Q8WYA6_CTBL1_HUMAN_CTNNBL1_C20orf33_PP8304.pdf]
